# Compact and modular autonomous surface vehicle for water research: The Naval Operating Research Drone Assessing Climate Change (NORDACC)

**DOI:** 10.1016/j.ohx.2023.e00453

**Published:** 2023-07-14

**Authors:** Daniel F. Carlson, Serkan Akbulut, Jeppe Fogh Rasmussen, Christian Søndergård Hestbech, Marius Hjorth Andersen, Claus Melvad

**Affiliations:** a Optical Oceanography, Institute of Carbon Cycles, Helmholtz-Zentrum Hereon, 21502 Geesthacht, Germany; b Department of Mechanical and Production Engineering, Aarhus University, Kathrinebjergvej 89, DK-8200 Aarhus N, Denmark; c Arctic Research Centre, Department of Biology, Aarhus University, Ole Worms Allé 1, DK-8000 Aarhus C, Denmark

**Keywords:** Autonomous surface vehicle, Surface water sample, Water quality

## Abstract

Research, monitoring, and management of marine and aquatic ecosystems often require surface water samples to measure biogeochemical and optical parameters. Traditional sampling with a boat and several personnel onboard can be labor-intensive and safety requirements limit sampling activities in high-risk environments. This paper describes the Naval Operating Research Drone Assessing Climate Change (NORDACC). NORDACC is an open source, light-weight, and portable autonomous surface vehicle that can acquire surface water samples while also measuring sea surface temperature and salinity for the duration of its deployment. NORDACC is ideal for operations in remote areas where resources and personnel are limited. Two sample bottles, each one liter in volume, can be filled, either at pre-programmed sampling stations or manually, using the remote control. A trimaran design provides buoyancy and stability, with hulls constructed of vacuum-formed acrylonitrile butadiene styrene (ABS) plastic. NORDACC can navigate autonomously between waypoints and features first person view capabilities for enhanced situational awareness. NORDACC’s performance was validated in Aarhus Bay, Denmark, collecting multiple surface water samples in winds in excess of 8 ms^−1^ and steep, choppy waves.

## Graphical abstract

**Figure:**
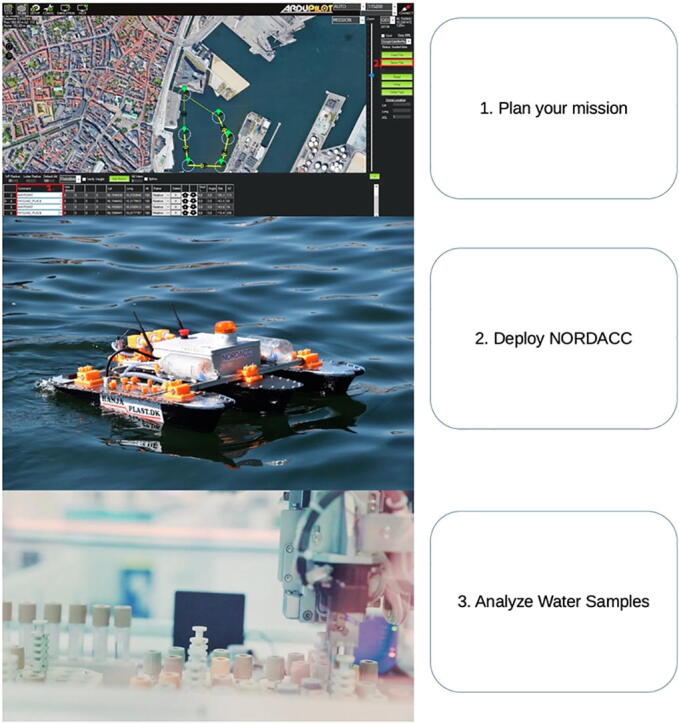

**Specifications table**

**Table:** 

**Hardware name**	NORDACC
**Subject area**	Environmental, planetary and agricultural sciences
**Hardware type**	Field measurements and sensors
**Closest commercial analog**	Autonomous Surface Vehicles
**Open source license**	CC BY 4.0
**Cost of hardware**	5500 €
**Source file repository**	https://doi.org/10.17632/rpzv35pccr.1

## 1 Hardware in context

Coastal habitats provide numerous functions and services, including, among others, supporting biodiversity and commercial and recreational fisheries and providing carbon storage [1]. Climate change and human activities threaten coastal ecosystems and the services that they provide to society [2]. Quantifying the degradation and aiding in the restoration of coastal ecosystems requires regular data collection [3]. Operating vessels in the shallow coastal zone can be challenging and sometimes dangerous due to highly variable weather and sea conditions, submerged hazards, and limited bathymetric data [4]. Data collection is especially challenging and dangerous near marine terminating glaciers and around icebergs [5]. The development of unoccupied marine robotic systems can, therefore, aid in monitoring and scientific studies and decrease the risk to humans [6].

Unoccupied surface vehicles (USVs) and autonomous surface vehicles (ASVs), hereafter referred to collectively as ASVs, have conducted a wide range of missions in marine and freshwater environments for at least several decades [5], [6], [7]. Our application required a small, affordable ASV that can collect discrete surface water samples and record continuous temperature and salinity measurements while operating in shallow water and in relatively harsh conditions. Commercially available ASVs either did not satisfy our requirements or were too expensive. Cheaper open-source platforms are also an option and some can be easily replicated without specific designs, parts or files [8], [9], [10], [11], [12] but they often have very specific operating requirements and few are designed to operate in harsh ocean conditions [13].

The Arctic Research Centre Autonomous Boat (ARCAB; [5], [14]) is a small ASV that successfully operated in harsh wintertime conditions in Greenland. This paper presents the Naval Operating Research Drone Assessing Climate Change (NORDACC), which improves upon the ARCAB design and introduces additional functionality, most notably the ability to collect surface water samples at pre-programmed waypoints. NORDACC was designed for use in hazardous environments in Greenland where traditional water sampling cannot be safely conducted. In particular, NORDACC was designed to collect surface water samples near the calving fronts of marine terminating glaciers, around icebergs, and in shallow, uncharted waters in Greenland. Greenland’s fjords are impacted to varying degrees by turbid meltwater runoff, which plays a critical role in marine ecosystems by limiting the light available for photosynthesis by primary producers like plankton and macrophytes [15]. The collection of surface water samples in these hazardous areas, therefore, will provide critical data for the calibration and validation of satellite-derived estimates of turbidity, chlorophyll and other parameters.

NORDACC shares some similar elements with the ARCAB project. Like ARCAB, NORDACC was a mechatronics student project that required a team of four bachelors students to design, build, and validate an autonomous vehicle that uses a National Instruments MyRIO coded with LabView. To reduce costs, NORDACC shares some electrical components with ARCAB and other student-built drones [16]. A retrospective analysis of the ARCAB project identified hull construction, modularity, communication, control, status indicators, and vehicle logs as areas for improvement. With regard to ARCAB’s construction, the use of moulds and fiberglass made the hulls difficult to replicate for those who lacked the experience, tools, and workspace necessary for fiberglass construction. Additionally a damaged or defective part of the ARCAB platform, particularly the hulls and the deck that connects them, are difficult to replace or repair.

## 2 Hardware description

NORDACC’s new design includes design improvements that were identified from a retrospective assessment of ARCAB. Furthermore, NORDACC introduces the new objective of acquiring surface water samples for water quality monitoring and the calibration and validation of satellite remote sensing water quality algorithms. Table 1 summarizes the requirements that guided the NORDACC design. (See Table 2, Table 3).

**Table 1:** NORDACC design requirements and comparison with ARCAB.

**Parameter**	**Requirement**	**NORDACC**	**ARCAB**
**1. Usage**			
Deployment	Ship or shore	X	X
Handling	1–2 people	X	X
Endurance	4 h	X	
Range	2 km	X	X
Speed	0.25–2 ms^−1^	X	X
Status Indicators	Color LED	X	
Visual Tracking	Strobe Light	X	X
**2. Water sampling**			
Sample Volume	⩾1 L	X	
No. Samples	⩾1	X	
Transfer Time	< 5 min	X	
Contamination	Flushable plumbing	X	
Collection	Waypoint and Manual	X	
Sample Depth	<15 cm below surface	X	
Sediments	Obtain sampling without clogging	X	
**3. Water column profiling**			
Acoustic Doppler Current Profiler	Mounting For ADCP	(X)	X
**4. Environment**			
Air Temperature	0 to 30°C	X	X
Water Temperature	0 to 20°C	X	X
Wind Speed	⩽15 ms^−1^	X	X
Waves	0.5 m	X	X
Current	0.25 ms^−1^	X	X
Salinity	Fresh to salt	X	X
**5. Design**			
Trim	Movable batteries	X	
Power	Hot-swap batteries	X	X
Assembly	Modular hull assembly	X	
Arm/Disarm	External switch	X	
Navigation Modes	Manual and Auto	X	X
Emergency	RC Override for MyRIO	X	
Turn Radius	<5 m	X	X
Directional Steering	Forward and Reverse	X	X
Situational Awareness	Real-Time Video (FPV)	X	
**6. Autonomy**			
Compatible with Mission Planner		X	X
Waypoint Navigation		X	X
Sampling Radius	⩽10 m while sampling	X	
Low Battery	Return to Home	X	
Moving Mother Ship	Dynamic Return to Home	X	
Obstacle Avoidance	Lidar system		X
Arctic Navigation	Dual GNSS	X	
**7. Data**			
Surface Data	Sea surface temperature and salinity	X	
Power	Transmit battery level	X	
Data Management	Log vehicle parameters and science data	X	
Log File	Vehicle mode, date/time, position, data	X	
**8. Transport**			
Shipping	Fits on euro pallet (80 × 120 cm)	X	X
Storage Temperature	−10°C to + 50°C	X	X

**Table 2:** An overview of the FrSky RC transmitter buttons and functions.

**Button**	**Function**	**Start position**	**Functionality by position**
Left joystick	Movement	Middle	Upwards: Forwards
	forwards/backwards		Downwards: Backwards
Right joystick	Movement	Middle	Right: Right
	right/left		Left: Left
SF	Controls manual/ autonomous mode	Downwards	Upwards: Autonomous mode
			Downwards: RC mode
SA	Manual water sample	Middle	Upwards: Fills right bottle
			Downwards: Fills left bottle
SG	Strobe on/off	Downwards/	When flicked upwards, and then back to start position strobe becomes
		Middle	If On → Off
			If Off → On
SD	Skip initialisation	Downwards/	If just started:
	Return to initialisation	Middle	
			Upwards flick: Skip initialisation
			
			If already initialised:
			
			Upwards flick: Return to initialisation phase

**Table 3:** An overview of the LED-indicator lights.

**Color**	**Flashing?**	**Description**
Blue	Yes	NORDACC is initialising → Components, GPS-position and mission
Purple	Yes	Sailing in autonomous mode
Blue	No	Sailing in RC mode
Green	Yes	Sampling
Red	Yes	Error: Low battery or water registered in pontoons

NORDACC simplifies hull construction by sourcing off-the-shelf vacuum-formed acrylonitrile butadiene styrene (ABS) plastic pontoons from Hanjaplast (https://hanjaplast.dk/) in cooperation with Billing boats (https://www.billingboats.com/). The ABS pontoons were chosen due to their low weight (750 g), strength and durability, and sharp angle of attack, which in turn reduces drag and increases the efficiency of the vessel. Each pontoon measures approximately 93×16×14 cm, with an internal volume of approximately 10L. Polyoxymethylene (POM) is used to seal the pontoons. A hatch in the middle of each pontoon, constructed from laser-cut acrylic (PMMA), permits easy inspection and access to the battery mount inside. 3D printed thumbscrews allow tool-less installation and removal of the hatches. In addition to holding the battery, the battery shelf design enhances the rigidity of the pontoon. Unlike ARCAB, which used a fixed catamaran hull, NORDACC uses a trimaran hull configuration. The hulls are connected using standard aluminum profiles and 3D printed brackets that allow the hulls to be attached and removed easily. NORDACC can be converted to a catamaran by removing the central pontoon. The pontoons can slide inwards to permit shipping on a euro pallet.

NORDACC uses two 1500 kV brushless motors in a skid-steering configuration. The motors are attached to the inside of the two outer pontoons by gluing 3D printed mounts to the inside of the hulls. Propellers are driven using a water-proof shaft. Two 3S 9000 mAh LiPo batteries connected in parallel provide power to the motors and all other electronics onboard NORDACC. LiPo decouplers prevent reverse current in the event that one battery fails or there being a difference in voltage between the batteries. Electronics are housed in a waterproof junction box. An external waterproof switch can be used to turn NORDACC on and off and can also disable the system in an emergency. Dual global navigation satellite systems (GNSS) receivers provide navigation unbiased by magnetic and geographical pole differences.

NORDACC carries two 1L sample bottles that can be filled at user-specified sampling waypoints or manually, by remote control. Waypoint missions can be planned using free and open source software like Mission Planner (https://ardupilot.org/planner/). Water samples are collected using a submerged pump and 3D printed manifold system that allows the plumbing to be flushed before storing each sample. A floating water level indicator in each sample bottle stops the pump automatically. The sample bottles can be installed and removed quickly and easily. In addition to collecting discrete water samples, NORDACC has two sensors that are mounted by the water pump to record the sea surface temperature (SST) and sea surface salinity (SSS). The SST and SSS are important indicators of gradients in coastal zones that are impacted by freshwater sources like rivers and glaciers.

During deployments, battery level, GPS coordinates, and status are transmitted to the ground station, which also displays the vehicle position on a live map. A DJI first-person-view (FPV) video system (https://www.dji.com/dk/fpv) transmits real-time video that shows the forward section of the NORDACC and its immediate surroundings. NORDACC uses a colored light emitting diode (LED) to indicate vehicle status. Different colors are displayed for initialization, manual operation, autonomous navigation, and water sample collection. The LED strip is visible in the FPV camera on NORDACC providing a second source of status information to the remote operator. NORDACC generates a log file for each deployment. The log file records the date, time, longitude, latitude, SST, SSS, and vehicle status. The log file is stored on a universal serial bus (USB) drive that is installed in the MyRIO.

NORDACC’s ground control system and autonomous navigation programs include fail safe procedures for both low battery power and water leakage. If either is detected, NORDACC will autonomously navigate to its home point. When operated from a moving boat, the real-time position of the ground station is used to guide NORDACC back.

• NORDACC uses modular ABS pontoons to simplify construction and maintenance.
• NORDACC allows discrete surface water samples to be collected, either at pre-programmed waypoints or manually using a remote control.
• NORDACC continuously records sea surface temperature (SST) and sea surface salinity (SSS) for the duration of each deployment. SST and SSS data are stored, along with date, time, and GPS coordinates (https://www.sparkfun.com/products/15136), in a detailed log file.
• NORDACC uses a first-person-view camera system to enhance situational awareness.

## 3 Design files summary

### 3.1 Hardware

.

**Table:** 

**Design filename**	**File type**	**Open source license**	**File Location**
001_ClampBasic.3MF	CAD	CC BY 4.0	Mendeley Data
002_ClampMidFront.3MF	CAD	CC BY 4.0	Mendeley Data
003_ClampMidBack.3MF	CAD	CC BY 4.0	Mendeley Data
004_ProfileBoxHolder.3MF	CAD	CC BY 4.0	Mendeley Data
005_ClampFingerScrew.3MF	CAD	CC BY 4.0	Mendeley Data
006_HatchFingerScrew.3MF	CAD	CC BY 4.0	Mendeley Data
007_HatchMount.3MF	CAD	CC BY 4.0	Mendeley Data
008_BoxPlatformPt1.3MF	CAD	CC BY 4.0	Mendeley Data
009_BoxPlatformPt2.3MF	CAD	CC BY 4.0	Mendeley Data
010_HullReinforceFrnt.3MF	CAD	CC BY 4.0	Mendeley Data
011_HullReinforceBck.3MF	CAD	CC BY 4.0	Mendeley Data
012_GNSSAntBase.3MF	CAD	CC BY 4.0	Mendeley Data
013_MotorMount.3MF	CAD	CC BY 4.0	Mendeley Data
014_Manifold.3MF	CAD	CC BY 4.0	Mendeley Data
015_ManifoldHoseAdapt.3MF	CAD	CC BY 4.0	Mendeley Data
016_ManifoldValveCover.3MF	CAD	CC BY 4.0	Mendeley Data
017_ManifoldRaiser.3MF	CAD	CC BY 4.0	Mendeley Data
018_CProfileClamp.3MF	CAD	CC BY 4.0	Mendeley Data
019_TorpedoPt1.3MF	CAD	CC BY 4.0	Mendeley Data
020_TorpedoPt2.3MF	CAD	CC BY 4.0	Mendeley Data
021_TorpedoPt3.3MF	CAD	CC BY 4.0	Mendeley Data
022_TorpedoPt4.3MF	CAD	CC BY 4.0	Mendeley Data
023_TorpedoPt5.3MF	CAD	CC BY 4.0	Mendeley Data
024_TorpedoPt6.3MF	CAD	CC BY 4.0	Mendeley Data
025_BatteryBridge.3MF	CAD	CC BY 4.0	Mendeley Data
026_SampleBottleNeck.3MF	CAD	CC BY 4.0	Mendeley Data
027_SampleBottleHolder.3MF	CAD	CC BY 4.0	Mendeley Data
028_SampleBottleConnector.3MF	CAD	CC BY 4.0	Mendeley Data
029_PontoonLidMiddle.3MF	CAD	CC BY 4.0	Mendeley Data
030_PontoonLidOuter.3MF	CAD	CC BY 4.0	Mendeley Data
031_HatchLid.3MF	CAD	CC BY 4.0	Mendeley Data

• The pontoon hulls are connected using aluminum profiles and 3D printed clamps: 001_ClampBasic.3MF, 002_ClampMidFront.3MF, 003_ClampMidBack.3MF.
• 004_ProfileBoxHolder.3MF holds the electrical box in place on the aluminum profiles.
• Finger screws are used to tighten the clamps and to close the access hatches: 005_ClampFingerScrew.3MF, 006_HatchFingerScrew.3MF.
• 007_HatchMount.3MF is used to hold the nuts, used for the hatch, in place.
• 008_BoxPlatformPt1.3MF and 009_BoxPlatformPt2.3MF are used for mounting electronics in the electrical box.
• 010_HullReinforceFrnt.3MF and 011_HullReinforceBck.3MF are installed inside the ABS pontoon hulls to reinforce the hull and to provide a mounting surface for the 025_BatteryBridge.3MF.
• The GNSS antennae are mounted on 012_GNSSAntBase.3MF.
• 013_MotorMount.3MF is used to mount each motor in the pontoon shells.
• The manifold in the water sampling system consists of four parts: 014_Manifold.3MF, 015_ManifoldHoseAdapt.3MF, 016_ManifoldValveCover.3MF, and 017_ManifoldRaiser.3MF.
• 018_CProfileClamp.3MF mount for the torpedo that allows the height of the torpedo to be adjusted.
• The submersible pump, temperature, and conductivity sensors are mounted in the ‘torpedo’, which consists of six parts: 019_TorpedoPt1.3MF, 020_TorpedoPt2.3MF, 021_TorpedoPt3.3MF, 022_TorpedoPt4.3MF, 023_TorpedoPt5.3MF, and 024_TorpedoPt6.3MF.
• 025_BatteryBridge.3MF is used to provide a flat surface, onto which the batteries can be mounted, this also allows for the batteries to be moved in order to change the trim of the entire vessel.
• The sample bottles are held in place using three parts: 026_SampleBottleNeck.3MF, 027_SampleBottleHolder.3MF, and 028_SampleBottleConnector.3MF.
• 029_PontoonLidMiddle.3MF lid for the middle pontoon with mounting holes only.
• 030_PontoonLidOuter.3MF lid with extra holes for wiring and access hatches for outer pontoons.
• 031_HatchLid.3MF is used for the access hatch lids.

### 3.2 Software

.

**Table:** 

**Software filename**	**File type**	**Open source license**	**File Location**	
Autonomous steering.vi	LabVIEW	CC BY 4.0	Mendeley Data	
Autonomous_direction_control.vi	LabVIEW	CC BY 4.0	Mendeley Data	
Battery charge.vi	LabVIEW	CC BY 4.0	Mendeley Data	
Battery level.vi	LabVIEW	CC BY 4.0	Mendeley Data	
BLDC.vi	LabVIEW	CC BY 4.0	Mendeley Data	
Command_library.vi	LabVIEW	CC BY 4.0	Mendeley Data	
Create data file.vi	LabVIEW	CC BY 4.0	Mendeley Data	
FrSky X8R.vi	LabVIEW	CC BY 4.0	Mendeley Data	
GPS_connection_validate.vi	LabVIEW	CC BY 4.0	Mendeley Data	
GPS_output_to_values.vi	LabVIEW	CC BY 4.0	Mendeley Data	
GPS.vi	LabVIEW	CC BY 4.0	Mendeley Data	
Initialise.vi	LabVIEW	CC BY 4.0	Mendeley Data	
Leakage sensor.vi	LabVIEW	CC BY 4.0	Mendeley Data	
LED indicator colour.vi	LabVIEW	CC BY 4.0	Mendeley Data	
Mission planner point select.vi	LabVIEW	CC BY 4.0	Mendeley Data	
Mission point.vi	LabVIEW	CC BY 4.0	Mendeley Data	
Motor power to direction.vi	LabVIEW	CC BY 4.0	Mendeley Data	
Motor_direction_control.vi	LabVIEW	CC BY 4.0	Mendeley Data	
Nordacc main.aliases	LabVIEW	CC BY 4.0	Mendeley Data	
Nordacc mainNordacc main	LabVIEW	CC BY 4.0	Mendeley Data	
Nordacc_main.vi	LabVIEW	CC BY 4.0	Mendeley Data	
Numerate_transmission_signals.vi	LabVIEW	CC BY 4.0	Mendeley Data	
Parse mission planner.vi	LabVIEW	CC BY 4.0	Mendeley Data	
Pump.vi	LabVIEW	CC BY 4.0	Mendeley Data	
Salinity sensor.vi	LabVIEW	CC BY 4.0	Mendeley Data	
sample tracker.vi	LabVIEW	CC BY 4.0	Mendeley Data	
Sampling system.vi	LabVIEW	CC BY 4.0	Mendeley Data	
Shared variables.lvlib	LabVIEW	CC BY 4.0	Mendeley Data	
Softstart.vi	LabVIEW	CC BY 4.0	Mendeley Data	
State_format converter.vi	LabVIEW	CC BY 4.0	Mendeley Data	
Strobe.vi	LabVIEW	CC BY 4.0	Mendeley Data	
Temp sensor coeffs.vi	LabVIEW	CC BY 4.0	Mendeley Data	
Temp_sensor.vi	LabVIEW	CC BY 4.0	Mendeley Data	
Time based boolean.vi	LabVIEW	CC BY 4.0	Mendeley Data	
Time based LED.vi	LabVIEW	CC BY 4.0	Mendeley Data	
Time based offset.vi	LabVIEW	CC BY 4.0	Mendeley Data	
USB_write_csv.vi	LabVIEW	CC BY 4.0	Mendeley Data	
Water leakage.vi	LabVIEW	CC BY 4.0	Mendeley Data	
Waypoint_percent.vi	LabVIEW	CC BY 4.0	Mendeley Data	
Wifi to variables.vi	LabVIEW	CC BY 4.0	Mendeley Data	
Wifi_recieve.vi	LabVIEW	CC BY 4.0	Mendeley Data	
Wifi_send.vi	LabVIEW	CC BY 4.0	Mendeley Data

• Autonomous steering.vi autonomously follows and keeps track of mission points, heading and position to output motor power that ensures the mission points are reached.
• Autonomous_direction_control.vi calculates heading of vessel, distance to mission point and the course offset.
• Battery charge.vi returns the lowest battery level and indicates if the battery level is critically low.
• Battery level.vi calculates battery percentage.
• BLDC.vi converts a power percentage [-100:100] to a PWM signal the motor can interpret.
• Command_library.vi outputs data in a delimited format [Name;value;value] to make it possible to save wifi data in variables.
• Create data file.vi creates a time and geo-stamped log file that shows the operation mode and event history.
• FrSky X8R.vi reads controller inputs and outputs. If RC is enabled, power percentage to the motors and when to sample in RC mode.
• GPS_connection_validate.vi checks if a GPS connection is established.
• GPS.vi reads and parses the NMEA $GNRMC message.
• Initialise.vi opens relevant communication ports on the MyRIO, parses the mission planner, creates a datafile and initializes shared variables.
• Leakage sensor.vi reads leakage sensor.
• LED indicator colour.vi controls the status LEDs.
• Mission planner point select.vi outputs the coordinates of the current mission point, or home destination if mission point exceeds mission length.
• Mission point.vi keeps track of the current mission point, and enforces return to home when mission is finished, or if water leakage or low battery is registered.
• Motor power to direction.vi converts degrees off target to power output for the two motors.
• Nordacc_main.vi main VI on NORDACC that is deployed when NORDACC is turned on, and the main functionality is based on a state machine.
• Numerate_transmission_signals.vi outputs a number to indicate the information that should be sent over WiFi.
• Parse mission planner.vi parses mission information from the onboard USB in the MyRIO. Mission points, and if they are sampling or waypoints.
• Pump.vi starts and stops the pump.
• Salinity sensor.vi reads the conductivity sensor output.
• Sample tracker.vi confirms if a sample has been filled.
• Sampling system.vi chooses the specific valve(s) that should be opened or closed to either flush or take samples and turns the pump on accordingly.
• Softstart.vi ensures no sudden direction change of the motors.
• Strobe.vi turns on or off the strobe.
• Temp sensor coeffs.vi uses the temperature sensor coefficients to convert the raw output of the temperature sensor to temperature in degrees Celsius.
• Time based boolean.vi when activated starts a countdown, and outputs remaining time of countdown and if it has finished.
• Time based LED.vi controls LED indicator’s on/off cycles.
• Time based offset.vi when a waypoint has been reached, it reduces the sensitivity to a course off set for a limited time.
• USB_write_csv.vi appends time stamp, sensor data and vehicle state to the log file created in the initialization.
• Water leakage.vi combines the values from both leakage sensors and outputs warning boolean.
• Waypoint_percent.vi calculates the distance traversed to the next waypoint in percent.
• Wifi to variables.vi saves data received from WiFi in variables.
• Wifi_recieve.vi outputs received WiFi string.
• Wifi_send.vi transmits data string over WiFi.

## 4 Bill of materials summary

The bill of materials is provided with the design files at: https://doi.org/10.17632/rpzv35pccr.1. Contact Billing Boats to purchase the vacuum-formed ABS hulls. (https://www.billingboats.com/).

## 5 Build instructions

### 5.1 3D printed and laser cut parts

Parts 7, 10, 11 and 25–30 (see Section 3.1) can be produced using a laser cutter or a computerized numerical control (CNC) router. The remaining parts, apart from the lower pontoons, can be produced using 3D printers. A print area of at least 200 mm × 200 mm × 150 mm is recommended (refer to the BOM and print instructions in the design files). PETG filament was used due to its chemical resistance and durability. Use clear nail polish, epoxy or waterproof paint to waterproof parts 14 and 15.

**Fig. 1:**
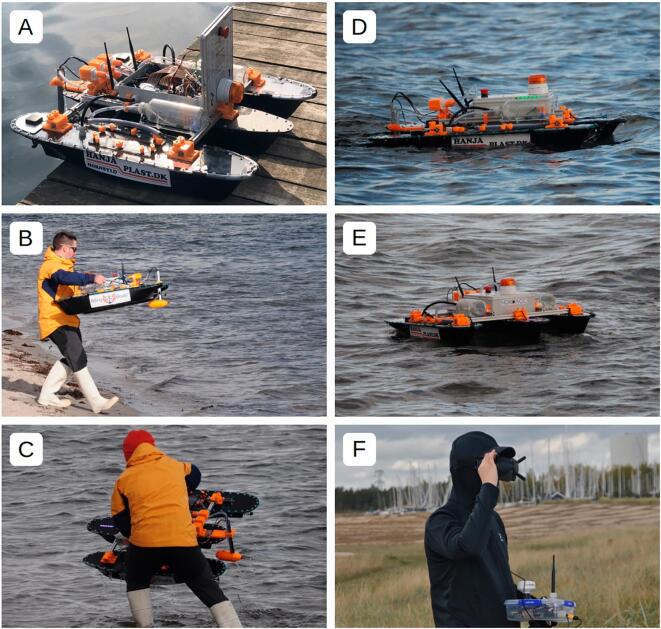
(A) Preparing NORDACC for deployment. (B) Single person deployment from a beach. (C) Single person retrieval from a beach. (D-E) Underway in choppy seas. (F) An operator viewing the live video stream from NORDACC using the FPV system. Also pictured is the ground station receiver.

### 5.2 Aluminum profiles

**Fig. 2:**
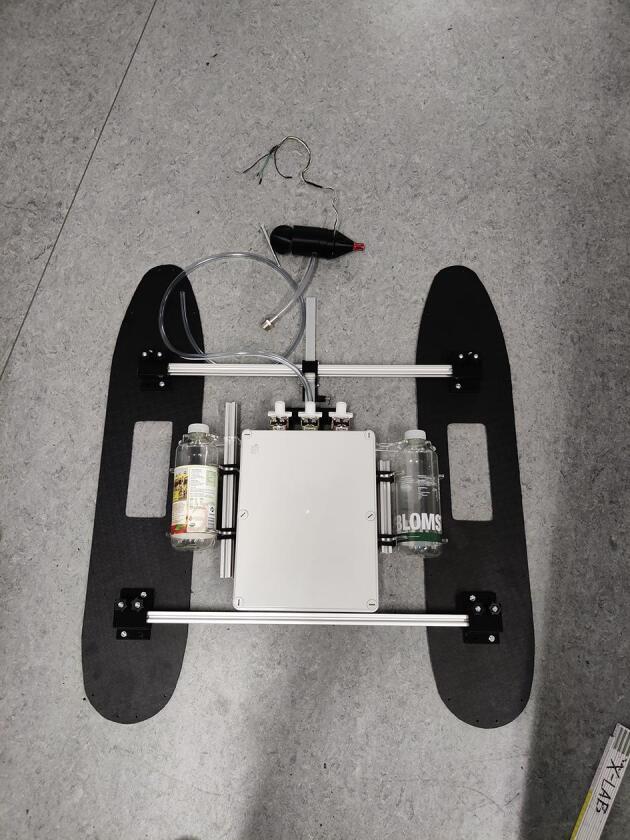
Aluminium profiles cut to length and initial mock up.

**Fig. 3:**
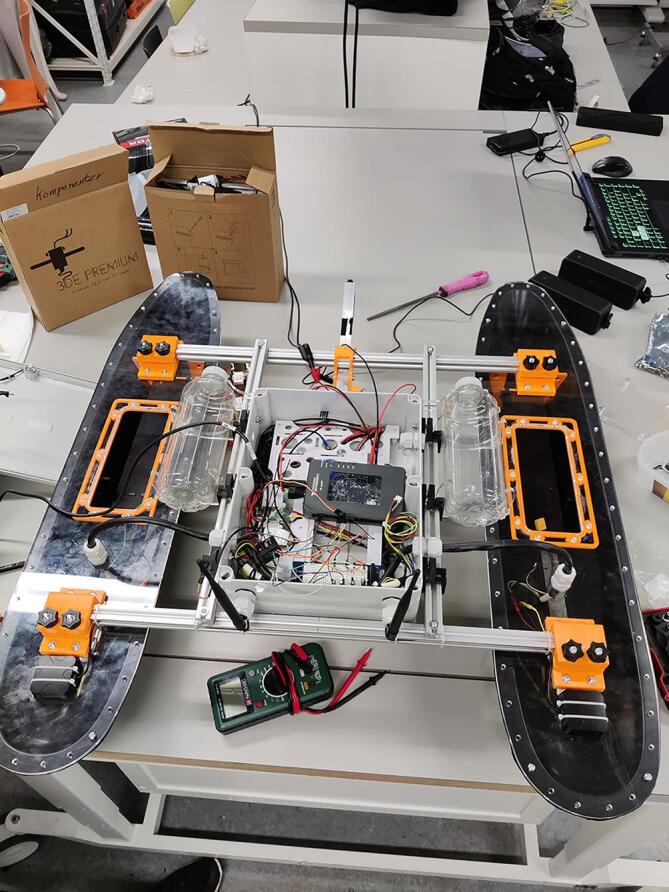
Main components fit together.

**Fig. 4:**
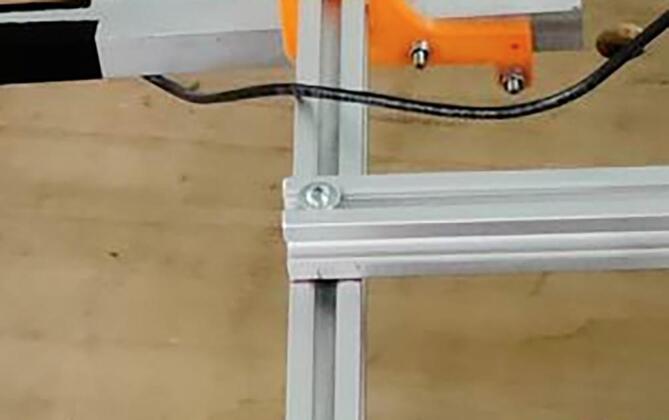
Aluminium profiles bolted together.

NORDACC uses 20 × 20 mm t-slot aluminum profiles to create a frame that connects the hulls and holds the electrical box and water sampling system. Cut four profiles for the frame, two with a length of 700 mm and two with a length of 550 mm (see Fig. 2, Fig. 3). Then drill four ∅4.5 holes in the top profiles where they match with the lower profiles (see Fig. 4).

### 5.3 Hull assembly

**Fig. 5:**
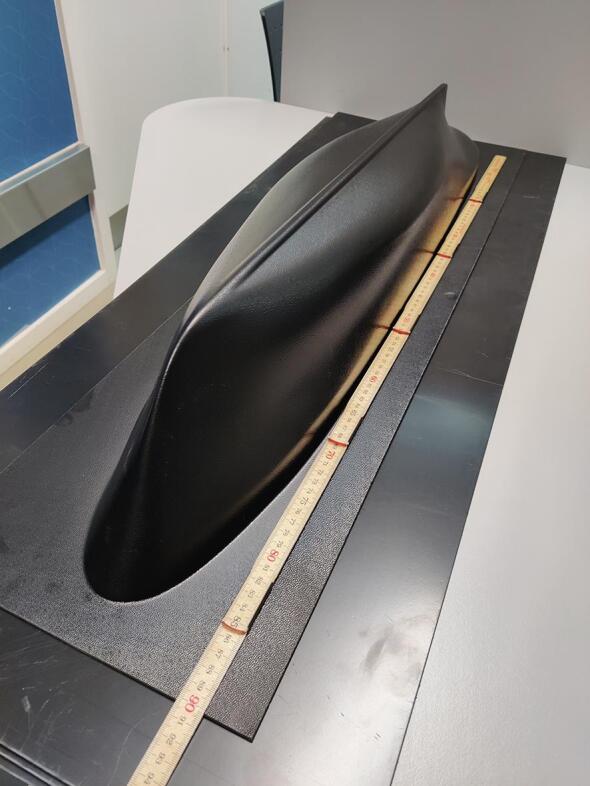
Vacuum-formed ABS pontoons as delivered from Hanja Plast.

**Fig. 6:**
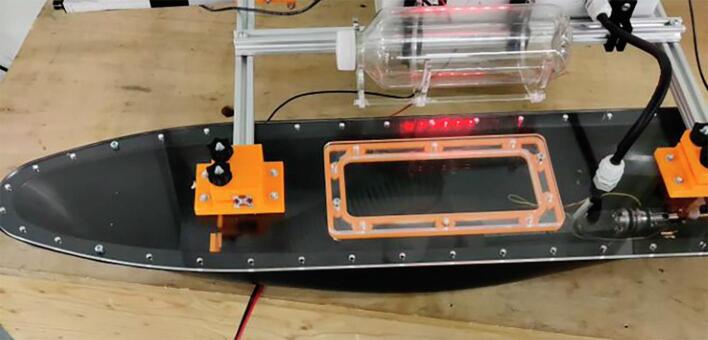
Lids and pontoons assembled.

The vacuum-formed ABS hulls (parts 33) must be trimmed to size and aligned with the top lid to create a water-tight seal. Align the hulls (see Fig. 5) with the lids (see parts 29 or 30 in the BOM), and drill 4–8 of the screw holes. Temporarily bolt the two parts together and use the pontoon lids as a template to score the flat portion of the pontoons with a utility knife; score the flat area several times until the plastic can be snapped off easily. Using the lids of the pontoons as template, drill the remaining holes along the edge for the bolts. (see Fig. 6).

Drill a hole in the rear of each pontoon for the propeller shaft to pass through. Pull all cables running between the electrical box and the pontoons through heat shrink tubing to ensure watertight seals at each end.

### 5.4 Electrical box

Use a drill with a step bit to make appropriate sized holes into the electrical box for the cable glands. Use hot glue in between the wire and shrink tubing around the wires where multiple wires are running into the electrical box to ensure that the cable glands will make a proper seal and no water will be able to leak through.

### 5.5 Sample bottles

.

**Fig. 7:**
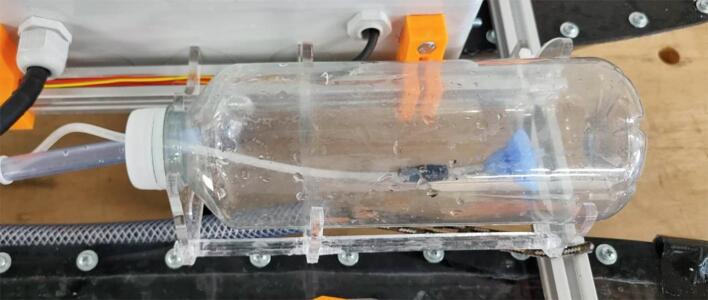
Air out system with weights and floaters.

1. If necessary, remove labels from the sample bottle using acetone.
2. Drill two holes in the lids of the sample bottle holders, one large for water in and one small for air out.
3. Installed pipes via press-fits in the drilled holes.
4. Two floaters made from foam pool noodles cut into approximate conical shapes 35 mm long and three M4 bolts attached to each air out pipe using heat shrink to ensure that the vent pipe is always on top of the water-sample to allow the air to exit as the bottle fills with water (Fig. 7).

### 5.6 Gluing

.

1. Press fits and super glue hold nuts and bolts to the parts, such as parts 5 to 7 (see Section 3.1).
2. Super glue parts 10, 11 and 13 in place.
3. Use a dissolved mixture of PMMA and acetone to glue parts 26–28 together.
4. Use silicone caulking to glue part 7 to allow for some flexing in part 30 during installation of the glued up assembly.
5. Use silicone caulking to ensure a watertight seal around the strobe and the LED strips.
6. To ensure a watertight seal between parts 30 and 31 (see Section 3.1) a custom rubber gasket was made; part 32.

### 5.7 Mounting

.

1. Mount the batteries to part 25 (see Section 3.1) using double sided velcro tape to allow the them to be moved forward or aft to adjust the trim of the boat.
2. Connect the 20×20 mm aluminium profiles using with bolts and T-nuts (see Fig. 3), in the previous drilled ∅4.5 holes.
3. Drill holes for the propeller shaft in the pontoon and seal with silicone caulking once the motor-propeller assembly is in place.
4. The propeller shafts can be either bought or made by hand. In this case, due to prices, a sleeve was made to fit on to the threaded shafts, to allow for a specific type of propellers made for RC boat racing.
5. Once the pontoons are finished they can be made watertight. Silicone caulking was used to make the center (empty) pontoon watertight. Electricians tape was used on the outer pontoons to reduce the disassembly time.

### 5.8 Electrical

**Fig. 8:**
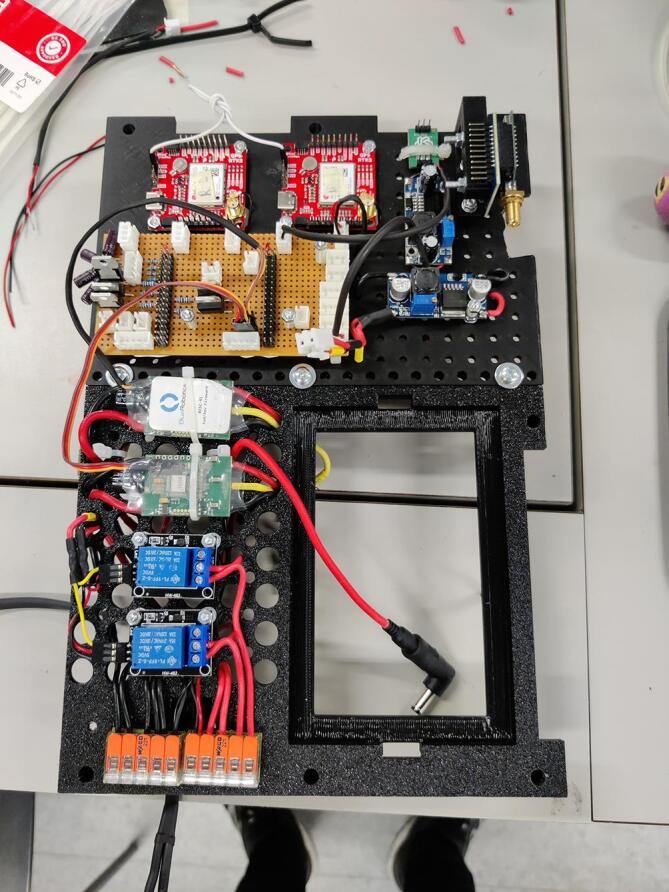
Shelf for components in the electrical box.

A veroboard was used for electrical connections between components (Electrical schematic). The PCB was mounted to the 3D printed box platform which was printed in two parts; parts 8 and 9. The two parts give a lifted perforated surface which the electronics could be fastened to by screws or zip ties. (Fig. 8).

**Optional parts**

1. A front bumper, made from rubber tubing, can be installed.
2. Simple handles, constructed from paracord and rubber tubing, can be installed to aid with deployment and retrieval (Fig. 1B,C).

## 6 Operation instructions

.

**Fig. 9:**
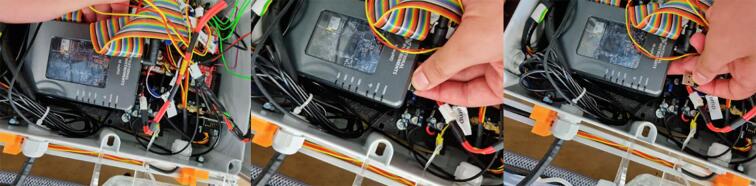
Open the electrical box and remove the USB drive from the MyRIO.
    1.2.2 Open Mission Planner.
    1.2.3 Navigate to ‘Plan mission’ tab, see the top left corner of Fig. 10.

1. **Pre-departure**
  1.1 Charge all batteries.
    1.1.1 9000 mAh 3s LiPo (at least 2; 4 batteries would allow for continuous operation if the batteries can be charged at the site).
    1.1.2 3s LiPo battery (For ground station; optional).
    1.1.3 FrSky RC transmitter.
    1.1.4 Laptop.
  1.2 Plan autonomous mission.
    1.2.1 Remove the USB drive from the MyRIO and plug it into your PC (Fig. 9).

**Fig. 10:**
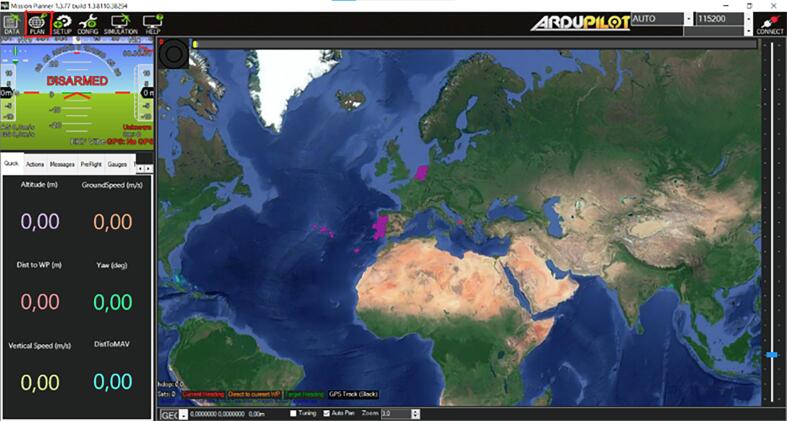
Mission Planner with the ’Plan’ icon outlined in red.
    1.2.4 Zoom into your region of interest.
    1.2.5 Define waypoints, see Fig. 11.

**Fig. 11:**
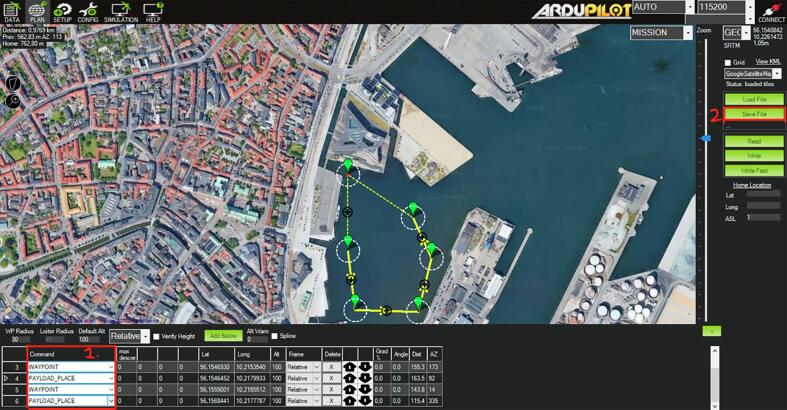
The command column (outlined in red) indicates the type of action that NORDACC should perform at each position. The WAYPOINT command instructs NORDACC to navigate to that position and then continue to the next waypoint. The PAYLOAD_PLACE command instructs NORDACC to collect a water sample at the specified location. It is not necessary to define a home position, because NORDACC will either return to the ground station, or its initial position if the ground station is not in use.
    1.2.6 Use the command ‘Payload place’ to define a water sample waypoint (Fig. 11). Note: Only two water sampling waypoints can be defined in a single mission.
    1.2.7 Save the mission on the USB drive using the file name ‘Mission.txt’.
    1.2.8 Insert the USB drive back into the MyRIO.
    1.2.9 Note: NORDACC creates a data file on the USB where sensor data, location and waypoint number are stored. The file is created as a csv-file with equal sign as the separator.
    1.2.10 Pre-fetch maps for offline use, if necessary.
  1.3 Check weather, sea conditions, boat traffic, local regulations, and location-specific hazards in the area of interest.
2. **Ground station setup (optional)**
  2.1 Connect the ground station battery.
  2.2 Connect the ground station to laptop via USB-cable.
  2.3 Open Labview project ‘Nordacc groundstation.lvproj’.
  2.4 Connect the laptop and MyRIO via Labview project.
  2.5 Start the programme ‘Ground station myrio.vi’.
  2.6 Start the programme ‘Nordacc groundstation main.vi’.
3. **FPV setup (optional)**
  3.1 Connect the battery to the FPV goggles, either through the ground station connection or directly to the ground station battery.
  3.2 Power on the goggles.
4. **NORDACC setup**
  4.1 Check the pontoons for water or evidence of previous leaks.
  4.2 Depress the switch located at the top of the electrical box.
  4.3 Plug in both batteries and charge connectors.
  4.4 Check for loose cables and cables near the motors.
  4.5 Seal the pontoons hatches using the finger screws.
  4.6 Before closing the electrical box, check for loose cables that could be pinched by the lid.
  4.7 Close and seal the lid of the electrical box.
5. **Launching NORDACC**
  5.1 Turn on the FrSky RC transmitter.
  5.2 Set the control switch to RC-mode.
  5.3 Turn on NORDACC by twisting the power switch on top of the electrical box.
  5.4 The ESCs should beep briefly.
  5.5 Check the LED-indicator. It should flash white and then blue to indicate that the system is initializing.
  5.6 Wait for the LED-indicator display a solid blue light.
  5.7 Ensure that the propellers can spin freely.
  5.8 Briefly move the left stick up and down to verify manual throttle control.
  5.9 Move the right stick from left to right to verify manual steering control.
  5.10 Lift NORDACC and place it gently into the water.
  5.11 Lower the ‘torpedo’ at the back of the middle pontoon, which contains the pump and the sensors, until it is fully submerged.
  5.12 Check the water sample hose connection.
  5.13 Ensure that the salinity sensor sticks out of the bottom of the torpedo.
  5.14 Use manual RC mode to steer NORDACC away from shore, while checking proper throttle and steering control.
  5.15 Activate the autonomous mode using the switch on the transmitter.
  5.16 Use the FPV goggles to verify that the LED-indicator flashes purple.
6. **Retrieving NORDACC**
  6.1 When NORDACC is close to the shore or the boat, switch to RC mode and steer it to the preferred pickup point.
  6.2 Ensure NORDACC is not moving or floating away before shutting off the power.
  6.3 Shut off the power to the motors by depressing the safety switch on the top of the electrical box.
  6.4 Raise the torpedo to the top position where it is above the bottom level of the pontoons.
  6.5 Gently lift NORDACC out of the water, and place it on a flat surface, making sure not to put on pressure the propellers, propeller shafts or the torpedo.
7. **Controller functionality overview**
  7.1 In RC mode, NORDACC can be steered forward/backward and left/right using the left and right gimbals, respectively (Fig. 12).

**Fig. 12:**
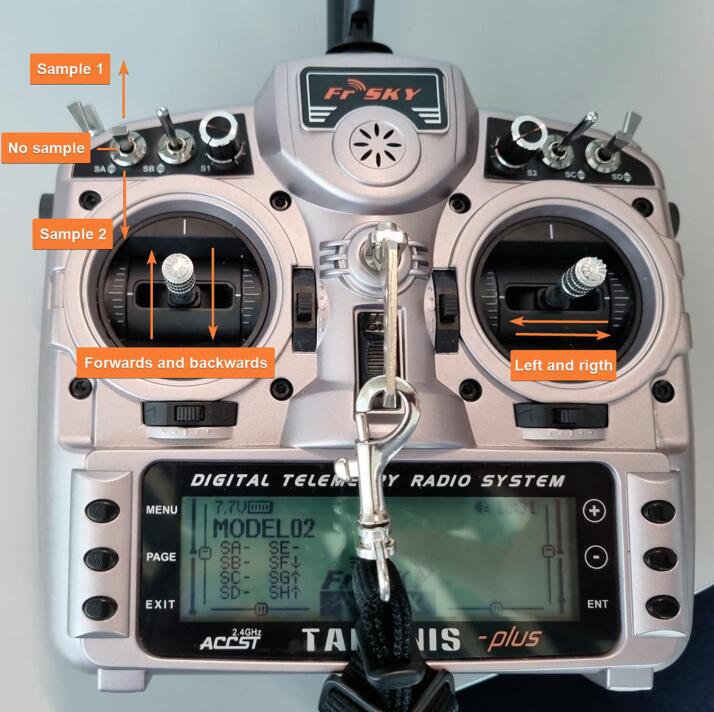
Taranis X9D controller with the functions of the gimbals and switches indicated.
  7.2 In RC mode, water samples can be collected using the 3-position SA switch on the top of the controller.
  7.3 The user can switch between RC and autonomous mode using the 2-position SF switch on the bottom-left side of the controller (when held facing away from the user; Fig. 13). Switching from autonomous mode to RC will disable all autonomous activities, including water sampling. (See Fig. 14)

**Fig. 13:**
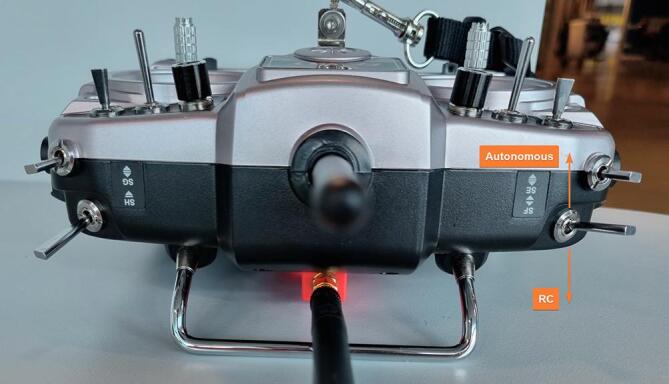
Controller from front.

**Fig. 14:**
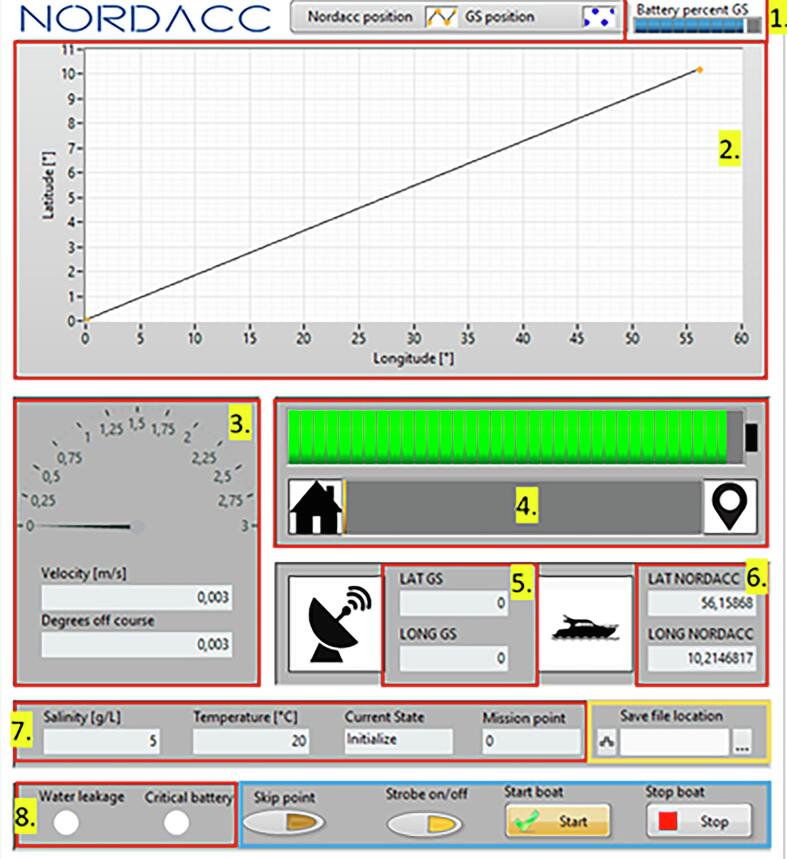
Ground station interface.
  **Note**, NORDACC is not controllable in RC-mode until the flushing and sampling has finished.
  **Upwards:** Controller seen from above, this is defined as away from the user. Controller seen from front, this is defined as down, following the orientation of the lettering.
  **Downwards:** Controller seen from above, this is defined as towards the user. Controller seen from front, this is defined as up, following the orientation of the lettering.
  **Flick:** Change switch position for 0.5 + seconds, and return it to start position.
  **SF:** If NORDACC is in autonomous mode when the mission is completed it returns to start location, or ground station if it is connected.
  **SA:** If the water sample is already filled NORDACC only flushes.
8. **LED indicator overview**
9. **Ground station interface functionality overview**

Software for NORDACC and the ground station are both written in LabVIEW (https://www.ni.com/da-dk/shop/labview.html).

Panels outlined in red display sensor information. Sensor inputs are described per their indexing number in the Figure above.

1. Battery percentage of ground station.
2. NORDACC’s coordinates and the relative position of the ground station.
3. NORDACC’s velocity and degrees off desired course (only in autonomous mode).
4. Battery percentage of NORDACC, and percentage travelled of distance between two waypoints.
5. Latitude and longitude of ground station.
6. Latitude and longitude of NORDACC.
7. Salinity, temperature, and what state is currently being executed (e.g sailing, sampling, and initializing). Lastly what point in the mission planner is currently executed. If this value is greater than the number of points in the mission plan an error has occurred or the mission has been completed. Both result in NORDACC returning to either the ground station or to its initial position based on deployment method.
8. Warning indicators: when these light up an error message is displayed. NORDACC automatically returns to home when these light up. This is also described in the error message.

The panel outlined in orange allow the user to define the file name where transmitted data is periodically saved (approximately every 30 s). It is also possible to check mission data from NORDACC’s on board USB post mission.

The buttons contained in the blue panel allow the user to interact with NORDACC, to either start or stop the boat and strobe, or skip waypoints in the mission.

## 7 Validation and characterization

**Fig. 15:**
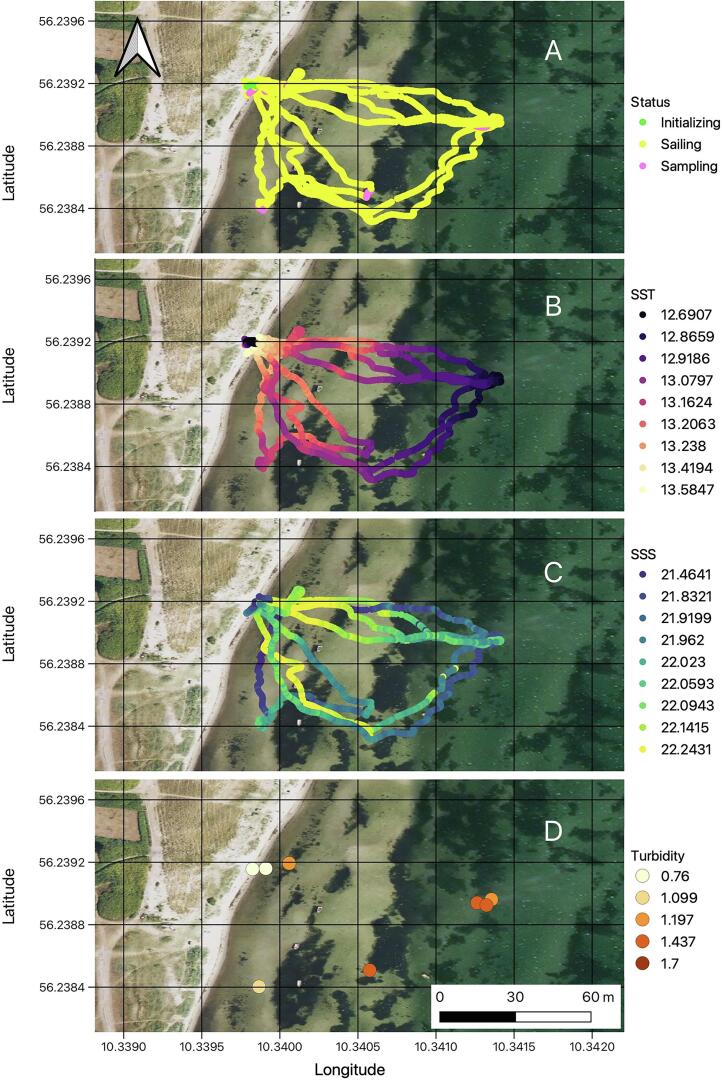
The data collected during the field validation show (A) vehicle status, (B) sea surface temperature (SST), (C) sea surface salinity, and turbidity (D).

NORDACC was originally conceived to assist with surface water sample collection in hazardous environments in Greenland. However, COVID-19-related complications did not permit tests in Greenland and demonstrations were conducted, instead, under similar wind and wave conditions in autumn in Denmark. Validation of NORDACC took place in Aarhus Bay on 14 October 2022. Environmental conditions were similar to those encountered in fjords in southwest Greenland in summer. Air temperature varied from 8–10°C, the wind speeds varied from 5–8 ms^−1^ with wave heights of approximately 0.5 m (Beaufort scale 3–4). NORDACC was pre-programmed to collect surface water samples at nearshore waypoints. Four missions were carried out and eight surface water samples were collected within the span of 90 min. NORDACC was launched and recovered from the beach by one person (Fig. 1). Surface water samples were collected at both pre-programmed waypoints and manually, using the remote control. Water samples were analyzed for turbidity using a Hach turbidimeter. Vehicle status and surroundings were monitored using the onboard FPV system. NORDACC positions, vehicle status, sea surface temperature (SST), sea surface salinity (SSS), and turbidity are shown in Fig. 15. Thus, the field validation of NORDACC demonstrates the ability to collect water samples for optical water quality parameters and to quantify surface gradients in SST and SSS. The validation data presented in Fig. 15 are available for download at https://zenodo.org/record/7990240 [17]. A short video description of the student project can be viewed at https://www.youtube.com/watch?v=KXrsCKIjEbk.

• Deployment, retrieval, and operation by a single person.
• Continuous measurements of sea surface temperature and conductivity.
• Autonomous or manual navigation.
• Collect 2 × 1L samples of surface water.
• Execute multiple sampling missions on a single set of batteries.
• Monitor vehicle status using a laptop ground station and/or FPV goggles.

## 8 Future improvements

While NORDACC performed well and met all of the design requirements in Table 1 we have identified areas that could be improved. The range of the FPV transmission system could be increased to provide the user with better manual control and overall situational awareness at greater distances by raising the antennae to a height of 0.75–1 m above the surface of the water. Object detection and collision avoidance could be added by installing a LiDAR or radar. Leakage sensors should be installed in the electronics box to better protect the electrical components. A waterproof USB access port could also be added, as the current placement of the USB drive on the MyRIO requires the electrical box to be opened, exposing its contents to the elements. A new manifold could be designed to increase the number of water samples that can be collected. Additionally, the sample bottles could be stored vertically to improve venting. The ground station display can be improved to indicate which sample bottle has been filled and which bottle is available and to transmit the battery voltage as well as the percentage remaining. NORDACC also has sufficient buoyancy to carry additional sensors. Future versions may include optical sensors to measure sea surface turbidity, chlorophyll, and chromophoric dissolved organic matter (CDOM) while the vehicle is underway. Additionally, radiometric sensors could be added, similar to the pySAS system [18].

## 9 Conclusions

We have described the design, construction, and validation of the Naval Operating Research Drone Assessing Climate Change (NORDACC). NORDACC operated autonomously in Egå (near Aarhus in Denmark) and continuously measured water temperature, water salinity and took several 1L water samples that were returned to shore for further analysis. This test demonstrated the potential for a small, portable, low-cost, and open-source ASV to enhance our understanding of coastal ecosystems.

## CRediT authorship contribution statement

**Daniel F. Carlson:** Conceptualization, Validation, Supervision, Funding acquisition, Writing - original draft. **Serkan Akbulut:** Writing - original draft. **Jeppe Fogh Rasmussen:** Writing - original draft. **Christian Søndergård Hestbech:** Writing - original draft. **Marius Hjorth Andersen:** Writing - original draft. **Claus Melvad:** Resources, Supervision, Funding acquisition, Writing - review & editing.

## Declaration of Competing Interest

The authors declare that they have no known competing financial interests or personal relationships that could have appeared to influence the work reported in this paper.
